# Case of Inflammation of the Liver, Terminating in Fatal Suppuration of the Organ, and Expectoration of Pus

**Published:** 1818-04

**Authors:** 


### *Case of Inflammation of the Liver, terminating in fatal Suppuration of the Organ, and Expectoration of Pus.* By Dr. Fouquier.

A Female domestic, aged 46, was admitted into the Charity-Hospital on the 18th of April 1817; and died on the 5th of June following.

This woman had never suffered from any other disease than the flooding consequent on several labours. An irregularity of the menstrual flux for eight months past, had seemed to announce its approaching cessation.

Six weeks previously to her admission into the hospital, she had been seized with colic, so violent as to induce insensibility. Dyspepsia, at the same time, prevailed. From the exhibition of a vomit and purgatives, some relief was obtained; and, at the expiration of a week, the woman resumed her occupation. Pain in the epigastric and right hypochondriac regions alone remained. She compared her sensations there to those of a wedge obstructing respiration. On the 23d of April, these pains were aggravated; and pressure on the parts which they occupied, still farther increased them. Fever and bilious vomiting supervened, with severe and dry cough; and, in the efforts which the latter provoked, some ropy mucus was ejected on the 24th. Respiration, in the mean time, continued difficult; the abdominal pains were unrelieved; and the urine assumed a brownish colour. On the 25th, after the evacuation of the bowels by an injection, some amendment was perceptible. In the evening, however, shiverings, succeeded by heat, came on; and the whole body assumed a jaundiced tinge. Such was the condition of the patient at the period of her entrance into the hospital. She could recline upon her back or on her left side, but not at all on the right. There was no head-ach; but she had flushing of the cheeks, a bitter taste in the mouth, thirst, loss of appetite; the tongue was loaded with a yellowish fur; and pain felt on pressure of the epigastric region and right hypochondrium. To these symptoms were joined flatulence, colic, and constipation, except when some liquid stools were procured by glysters. Tbe patient coughed and expectorated a little transparent frothy mucus. Percussion of thechest excited no pain; and the cavity sounded well notwithstanding the tightnes of respiration. The pulse was full and frequent: the skin hot and dry; but there existed a perfect integrity in the exercise of the senses. Blood-letting to the amount of ten ounces, an anodyne julep, injections, and sparing diet, were now prescribed, Evident relief succeeded the loss of blood. The pain and pyrexia were diminished; and the urinary secretion less high-coloured than before. On the evening of the 27th, shivering recurred, and was succeeded by heat and perspiration. The pain of the hypochondrium was much increased; and, after fruitless efforts to vomit, some bile was evacuated by a glyster: yet a little sleep was procured. There was some remission of the principal symptoms; but they returned towards evening. A poultice on the epigastrium was added to the former treatment. As pain and fever continued on the 29th, I prescribed bleeding anew. But the jaundice still increased, and there was invariably a febrile exacerbation in the evening. Some drowsiness was experienced during this night and on the morning of the 30th ; a diminution of the epigastric and hypochondriac pains, however, followed the discharge of four liquid stools; and the evening exacerbation was less considerable. On the 1st of May, there were great debility and exhaustion, with frequent nausea and vomiting of a small quantity of yellow fluid, and colic during the night. Narcotics were now added to the epigastric cataplasm. Until the 10th of May, with the exception of a slight cough, and occasional vomiting, a general amendment was observed; but, at that time, an acute pain attacked the lower part of the right side of the thorax: respiration became short and painful; and some viscid fluid was with difficulty expectorated. The pulse was hard, wiry, and frequent. Fifteen leeches, and subsequently an emollient cataplasm, were applied to the seat of pain. On the morrow, the pulse was feeble and intermittent; and the right side of the chest painful on percussion, and obscure in sound, particularly at the inferior part. Leeches were again applied. For some days, the cough was relieved and expectoration less difficult; but the right side of the chest invariably rendered an obscure sound. On the 18th, the pulse was again accelerated; the heat increased; and respiration become more laborious. Fifteen leeches were applied beneath the right axilla; but there was still a harassing cough, with copious expectoration. The fever subsided on the 24th; but, two days afterwards, was, with all the other symptoms, exasperated. In the night of the 1st of June, considerable oppression came on with frequent cough, and expectoration more abundant and opaque than ordinary. A greenish bitter fluid was also rejected by vomiting. I now prescribed a grain of tartrite of antimony; and, after a discharge of bile induced by it, a yellowish, fœtid, puriform fluid was copiously expectorated. On the 3d, there was evidently much depression; yet the respiration was more free. Expectoration the same as on the preceding night. The pulse, on the 4th, was small and feeble; and obscure fluctuation was perceptible in the abdomen. The oppression was also very considerable, and the forces broken. I now prescribed the application of a large blister to the thorax, and the internal use of cinchona. But the patient died during the night.

*Dissection.* The body presented externally the appearances of marasmus aud jaundice. The interior of the cranium was not examined. After having cautiously opened the thorax, I introduced air into the trachea by means of a blow pipe; and the gurgling and escape of bubbles that ensued, bespoke a communication between the right lung and cavity of the corresponding pleura. Into this cavity a large quantity of purulent yellowish serum was effused, possessing the odour of rancid bacon. The right lung, reduced to one-third of its ordinary size, adhered to the diaphragm by its base; and presented, in this place, an erosion corresponding to an opening in the diaphragm which was capable of receiving the finger. The structure of this lung was indurated, destitute of air, and partly injected with reddish serum. A false membrane enveloped this viscus, and lined the corresponding pleura. The left sac of the pleura contained some ounces of reddish serum, and presented also adhesions formed by false membrane. The lung of this side, compressed by the fluid, was gorged with a reddish frothy serum. The abdominal cavity contained no fluid. The liver possessed no unusual volume; although its structure was more red and firm than ordinary. On the convex surface of its right lobe, was seen a cavity capable of containing a pullet's egg, and half-full of yellow and highly offensive pus. The superior paries of this cavity was formed by the diaphragm. In the point corresponding to this abscess, was presented the perforation in the diaphragm above-mentioned. The gall-bladder was filled with a whitish puriform fluid. Its parietes, like all the other parts of the abdomen, were sound.*

* *Bulletin de la Faculté de Médecine de Paris,* &c. 1817—No. VI.

